# Longitudinal Trajectories of Grief Before and After Bereavement and Their Association With Mental Health Outcomes: A Pilot Prospective Cohort Study From the China Grief Cohort 2025

**DOI:** 10.1155/da/5274616

**Published:** 2026-09-28

**Authors:** Chunfeng Xiao, Song Wan, Xinpeng Xu, Wentao Yao, Yufei Wang, Jing Wei

**Affiliations:** ^1^ Peking Union Medical College Hospital, Chinese Academy of Medical Sciences and Peking Union Medical College, Beijing, China, cacms.ac.cn; ^2^ Universal Scientific Education and Research Network (USERN), Beijing, China, usern.org; ^3^ USERN China Office (USERN Scientific Collaboration Center, USCC), Beijing, China; ^4^ Department of Psychiatry, Suzhou Minkang Hospital, Suzhou Social Welfare General Hospital, Suzhou, Jiangsu Province, China; ^5^ Beijing Huilongguan Hospital, Capital Medical University, Peking University Huilongguan Clinical Medical School, Beijing, China, ccmu.edu.cn

## Abstract

**Background:**

Prolonged grief disorder (PGD) impairs psychophysiological well‐being and social functioning. Although post‐death grief trajectories have been well‐documented, pre‐death grief trajectories remain understudied, particularly within the same cohort.

**Objective:**

This exploratory pilot study aimed to identify candidate longitudinal trajectories of grief before and after bereavement and to examine their associations with subsequent mental health outcomes, with the goal of generating hypotheses to inform a larger confirmatory cohort.

**Methods:**

This prospective cohort study was conducted at Peking Union Medical College Hospital. Participants were adult immediate family members of terminally ill patients with a life expectancy of 6 months or less enrolled between October and December 2023. Grief, depression, anxiety and stress were measured using the Anticipatory Grief Scale (AGS) and the PGD‐13 Revised (PG‐13‐R), the 17‐item Hamilton Rating Scale for Depression (HAMD‐17), the Hamilton Anxiety Scale (HAMA) and the Impact of Event Scale‐Revised (IES‐R), at baseline, monthly before bereavement and quarterly for 6 months after bereavement, respectively.

**Results:**

A total of 60 participants (mean [SD] age, 52.82 [10.18%] years; 32 [53.3%] women) were enrolled, most were offspring of terminally ill patients (44 [73.3%]). Two pre‐death grief trajectories (Low, *n* = 51 [85%]; and increasing, *n* = 9 [15%]) and four post‐death grief trajectories (high/decreasing, *n* = 9 [15%]; moderate/decreasing, *n* = 26 [43.33%]; low/decreasing, *n* = 15 [25%]; and high/prolonged, *n* = 10 [16.67%]) were identified. At the final visit, the incidences of PGD, depressive disorders, anxiety disorders and stress‐related disorders were 16.7%, 16.7%, 30.0% and 0%, respectively. Participants in the Increasing pre‐death grief trajectory and the persistently high/prolonged post‐death grief trajectory had higher incidences of PGD, depressive disorders, and anxiety disorders compared to other groups.

**Conclusions:**

Individuals experiencing increasing pre‐death grief or persistently high‐level post‐death grief are more likely to develop PGD and other mental disorders. Findings are exploratory and require replication in a larger, theoretically informed cohort.

**Trial Registration:** Chinese Clinical Trial Registry Identifier: ChiCTR2300071236

## 1. Introduction

Grief encompasses a range of psychophysiological responses experienced by individuals following the loss of a loved one. As research in this field has advanced, the conceptualisation of grief has evolved, with increasing academic focus on understanding its developmental trajectories and the implications for broader populations. Singer et al. [1] systematically reviewed existing concepts and definitions related to grief, highlighting pre‐death grief as a critical area of scholarly inquiry. Pre‐death grief is comprised of two distinct types of grief responses: anticipatory grief and illness‐related grief [1]. Anticipatory grief is future‐oriented and involves separation distress and concerns about a future without the individual with a life‐limiting illness. In contrast, illness‐related grief is present‐oriented and characterised by grief over current and ongoing losses experienced during the illness trajectory. Post‐death grief, previously defined as grief in its conventional sense, manifests in various forms, including acute, integrated and complicated grief. While most grief reactions are considered normative responses and typically do not require clinical attention, some atypical forms of grief can severely impair health and social functioning [2]. Bolby [3] emphasised the importance of recognising abnormal grief responses. Prolonged grief disorder (PGD) has been formally recognised as an independent diagnosis by both the DSM‐5‐TR and ICD‐11. PGD is characterised by persistent and severe emotional, cognitive and behavioural distress following bereavement. Cognitive symptoms include persistent and intense yearning for the deceased, preoccupation with thoughts or memories of the deceased, and difficulty accepting the loss; emotional symptoms include intense emotional pain (e.g., sadness, guilt and anger), feelings of disbelief, and a sense of meaninglessness; and behavioural symptoms include avoidance of reminders of the loss, social withdrawal, and difficulty re‐engaging in activities or relationships. These features distinguish PGD from normative grief reactions, which are typically self‐limiting and do not produce sustained functional impairment. These symptoms extend beyond culturally, socially or religiously expected timeframes, significantly impairing social and occupational functioning and necessitating clinical attention [4, 5]. Although PGD is formally classified as a stress‐related disorder, it is differentiated from other stress‐related conditions by its core symptom of persistent yearning and preoccupation with the deceased. A meta‐analysis estimated the pooled prevalence of PGD at ~9.8% in non‐psychiatric adult populations exposed to non‐violent bereavement, with prevalence rising to ~50% among bereaved individuals following unnatural or violent losses [6, 7], underscoring its substantial public health burden.

From a clinical phenomenological perspective, excluding cases of sudden death, the transition from pre‐death grief responses to PGD constitutes a dynamic process unfolding over time rather than a series of discrete stages or a static cross‐sectional spectrum. We previously proposed the PGD developmental atlas, a model intended to unify grief‐related concepts and definitions, which qualitatively describes the longitudinal patterns of grief responses and offers preliminary insights into PGD pathogenesis and progression with the aim of creating a theoretical foundation for future quantitative studies [8]. Briefly, the developmental atlas posits that grief responses unfold along a temporal continuum spanning the pre‐death and post‐death periods. In the pre‐death period, family members typically experience escalating anticipatory and illness‐related grief as the patient’s condition deteriorates, with substantial inter‐individual variability in the rate and magnitude of escalation. The death itself constitutes an inflection point, after which trajectories diverge: most individuals exhibit gradual recovery (resilient or integrated grief), whereas a minority exhibit persistently elevated grief that meets PGD criteria. Pre‐death grief intensity, the relationship to the deceased, and circumstances of the illness or death are hypothesised to shape post‐death trajectories. The empirical findings of the present study can be mapped onto this framework, with the persistently low‐level pre‐death and low/decreasing post‐death trajectories representing the resilient pathway, the increasing pre‐death and high/prolonged post‐death trajectories representing the at‐risk pathway towards PGD, and the intermediate trajectories representing transitional patterns. A limited number of longitudinal studies had preliminarily investigated PGD trajectories before the previously mentioned framework. Existing research has predominantly focused on post‐death grief, with scarce attention given to pre‐death grief. These studies have been conducted in adult bereaved populations using a range of measurement tools and follow‐up windows. For instance, one of the earliest longitudinal studies on grief trajectories analysed PG‐13 scale scores at pre‐loss, 6 months post‐loss, and 3 years post‐loss using the Caregiver Cohort 2012 database and finally identified five distinct grief trajectories among bereaved individuals who had lost a partner: low, moderate/decreasing, high/decreasing, high, and late grief [9]. The late‐grief trajectory was exclusively observed among individuals bereaved of a partner and not those who lost non‐partners. More recently, Lundorff et al. [10] conducted a cohort study involving 857 widowed individuals over 11 months. Their analysis identified four grief trajectories: resilient, moderate‐stable, recovery and prolonged. Individuals in the prolonged grief trajectory exhibited a higher propensity for developing PGD, with correlates including low optimism and poor mental health status. Additional studies have assessed not only grief but also associated psychiatric symptoms, such as depression and stress [11–13]. Beyond the characteristics of the bereaved individual, evidence indicates that the manner and circumstances of the death exert a substantial influence on the course of grief. Adult studies have shown that violent and unnatural losses (e.g., suicide, homicide and accidents) are associated with markedly elevated rates of PGD compared with anticipated deaths from natural causes [14], and qualitative work suggests that witnessing prolonged suffering or distressing physical symptoms during the dying process can intensify pre‐death grief and complicate post‐death adjustment. A mixed‐methods study further demonstrated that emotion‐oriented coping strategies were associated with lower pre‐death grief intensity, whereas dysfunctional coping strategies were associated with higher grief levels among family carers [15]. The dying process can also introduce or exacerbate a broad array of secondary stressors, including caregiving burden, financial strain, and disruption of family relationships, that may independently shape grief outcomes. These observations underscore the importance of including measures of stressful life events as a potential correlate, predictor, and covariate in trajectory studies. However, variations in study environments, measurement tools, and methodologies have resulted in inconsistent findings across studies. Most follow‐ups started only after bereavement, resulting in a significant gap in our understanding of the longitudinal patterns of pre‐death grief and its transition to PGD. This lack of data limits the empirical foundation needed to identify candidate correlates and predictors of high‐risk trajectories that may, in turn, inform risk screening, prognosis, and theoretical refinement of PGD pathogenesis.

This study aimed to examine the dynamic changes and longitudinal trajectories of grief during the pre‐ and post‐death periods and their association with subsequent mental disorders, including but not limited to PGD. Additionally, it explored the longitudinal trajectories of other key psychiatric symptoms (i.e., depression, anxiety and stress) in order to identify candidate factors that may populate conceptual frameworks of grief development. Given the modest sample size and the absence of a replication cohort, this study is explicitly framed as exploratory: its purpose is to generate hypotheses regarding correlates and predictors of grief trajectories that can be tested in larger, theoretically informed studies, rather than to support causal inference or to inform specific intervention strategies.

Building on the literature reviewed above, this exploratory pilot study addressed five inter‐related questions: (a) Can distinct longitudinal trajectories of grief be identified during the pre‐death and post‐death periods using group‐based trajectory modelling (GBTM) in a Chinese cohort of family members of terminally ill patients? (b) Are pre‐death grief trajectories associated with post‐death grief trajectories, and are these grief trajectories associated with the trajectories of related but distinct psychiatric symptoms (depression, anxiety and stress)? (c) What demographic, relational and clinical characteristics correlate with membership in different grief trajectories, particularly factors related to the bereaved individual (e.g., relationship to the patient and emotional closeness), the dying process (e.g., manner of death, estimated remaining life expectancy and organ system of the terminal condition) and the surrounding stressor context (life events)? (d) What proportion of participants meet dignostic criteria of mental disorders of interest at the end of follow‐up and how do these rates differ across the identified trajectories? (e) Do the patterns observed in this Chinese terminal‐illness cohort align with the previously proposed PGD developmental atlas and with adult bereavement studies conducted in Western populations? These five questions are tracked systematically through Section 3 and Section 4.

## 2. Methods

### 2.1. Trial Design

This is a pilot study for the China Grief Cohort 2025; an exploratory, single‐centre, prospective cohort design was selected to assess feasibility and to generate hypotheses for the larger main cohort. The study was conducted at Peking Union Medical College Hospital. The authors assert that all procedures contributing to this work comply with the ethical standards of the relevant national and institutional committees on human experimentation and with the Helsinki Declaration of 1975, as revised in 2013. This study was approved by the Ethics Committee of Peking Union Medical College Hospital (Approval Number I‐23PJ713). All participants provided written informed consent before enrolment. The study followed the Strengthening the Reporting of Observational Studies in Epidemiology (STROBE) reporting guideline.

### 2.2. Patients

The inclusion criteria included individuals aged ≥18 years who are immediate family members of terminally ill patients with a life expectancy of ≤6 months and were informed of the imminent death of the terminally ill patient. The immediate family member was defined as the spouse, parent, child, adoptive parent, or adoptive child of the patient or any individual who had lived with the patient for ≥5 years in the specified order of priority. To minimise within‐family clustering and to ensure the independence of observations, recruitment was limited to one immediate family member per family. When more than one eligible family member was available within a family, the family member with the highest priority was approached first (priority order: spouse, parent, child, adoptive parent, adoptive child and cohabitant of ≥5 years). If the highest‐priority family member declined or was ineligible, the next family member in the priority order was approached. The exclusion criteria included the following: (1) diagnosed with severe illness who were aware of their own impending death; (2) a history of any mental disorders, e.g., major depressive disorder; (3) at a high risk of suicide.

### 2.3. Assessments

Participants were assessed at baseline, monthly during the pre‐death period, and every 3 months for 6 months during the post‐death period. Grief symptoms were evaluated using the Anticipatory Grief Scale (AGS; 27–135, with higher scores indicating higher levels of anticipatory grief) before bereavement and the PGD‐13 Revised (PG‐13‐R; range, 10–50, with higher scores indicating higher levels of post‐death grief) after bereavement [16, 17]. Depressive symptoms were assessed using the 17‐item Hamilton Rating Scale for Depression (HAMD‐17; range: 0–52, with higher scores indicating more severe depressive symptoms). Anxiety symptoms were evaluated using the Hamilton Anxiety Scale (HAMA; range, 0–56, with higher scores indicating more severe anxiety symptoms), and stress symptoms were assessed using the Impact of Event Scale‐Revised (IES‐R; range 0–88, with higher scores indicating more severe stress symptoms). The Columbia‐Suicide Severity Rating Scale (0–42; higher score indicating greater suicidal risk) was rated throughout the trial for safety. We included an evaluation of life events using the life event scale (LES) to minimise the potential confounding influence of other positive or negative life events on the mental health state of the participants during the study period, thus preserving the integrity of the primary mental health outcomes [18]. Emotional closeness between the participant and the terminally ill patient was assessed at baseline using the VAS‐Closeness Scale, a single‐item visual analogue scale ranging from 0 to 10. The scale anchors are a score of 0 indicates ‘I have minimal emotional connection with the family member who is about to pass away; his/her departure has no impact on me, and compared with the love I have for my most beloved person, my love for him/her is almost negligible’ and a score of 10 indicates ‘I have a deep emotional connection with the family member who is about to pass away; I cannot imagine how I would cope without him/her, and compared with the love I have for my most beloved person, my love for him/her is equal to or even greater’. Higher scores indicate greater emotional closeness. Medically estimated remaining life expectancy of each terminally ill patient was determined by the attending physician at study enrolment, on the basis of the patient’s primary diagnosis, disease stage, response to prior treatments, and current functional status and recorded in months. All symptom scales above (AGS, PG‐13‐R, HAMD‐17, HAMA and IES‐R) were used as dimensional measures of symptom severity over time for the purpose of trajectory modelling. They were not used to define the diagnostic caseness. Instead, at the end of follow‐up (6 months after bereavement), each participant underwent a clinical diagnostic interview conducted independently by two senior psychiatrists, both of whom were blinded to the participant’s trajectory group membership and to each other’s ratings. Diagnoses of PGD and other mental disorders were made according to the corresponding ICD‐11 diagnostic criteria. A participant was considered a confirmed case only when both psychiatrists independently arrived at the same diagnosis; any discrepancy was resolved by review with a third senior psychiatrist (Jing Wei). This two‐rater consensus procedure was adopted to maximise diagnostic specificity in a population where culturally normative expressions of grief could otherwise inflate apparent caseness on self‐report instruments alone.

### 2.4. Outcomes

Our primary outcomes were changes in AGS and PG‐13‐R scores and specific trajectories of grief before and after bereavement. Secondary outcomes included changes in scores on the HAMD‐17, HAMA and IES‐R, as well as specific trajectories of psychiatric symptoms, including depression, anxiety, stress and incidence of PGD and other mental disorders. The incidence of these disorders was established by a two‐rater clinical diagnostic interview using ICD‐11 diagnostic criteria, with diagnoses confirmed only when both psychiatrists independently agreed. The symptom scales were used as dimensional inputs to the trajectory analyses described below and not as the basis for diagnostic classification.

### 2.5. Statistical Analysis

Normally distributed variables are presented as mean ± standard deviation (SD; x̅ ± s) and compared using independent‐sample *t*‐tests (two groups) or one‐way analysis of variance (ANOVA; multiple groups). Non‐normally distributed variables are presented as a median and interquartile range (M [P25, P75]) and compared using the Mann–Whitney *U* test (two groups) or the Kruskal–Wallis *H* test (multiple groups). Categorical variables are presented as frequency and percentage (%) and compared using the *χ* ^2^ test. GBTM was used to identify latent clusters of participants with similar trajectories. To avoid tautology when subsequently characterising trajectory groups, GBTM was applied separately to each repeated symptom score: pre‐death AGS scores were used as the sole input to derive pre‐death grief trajectories; PG‐13‐R scores from post‐death assessments were used as the sole input to derive post‐death grief trajectories; and HAMD‐17, HAMA and IES‐R scores were used as the sole inputs to derive their respective depression, anxiety and stress trajectories at pre‐death and post‐death. All other variables, including demographic characteristics, the relationship to the terminally ill patient, the VAS‐Closeness Scale, the medically estimated remaining life expectancy, the primary diagnostic system classification, life events on the LES and rates of subsequent mental disorders, were used only for characterising the derived trajectory groups and were not entered into the GBTM models. The reported correlation between pre‐death (AGS‐derived) and post‐death (PG‐13‐R‐derived) grief trajectories therefore reflects the association between latent groups derived from non‐overlapping variable sets rather than a methodological artefact. The developmental trajectories of pre‐death grief were examined at 3, 2 and 1 month(s) before bereavement (i.e., the 3 monthly assessments closest to the death). This three‐assessment window was chosen a priori for two reasons. First, all enrolled patients had a medically estimated life expectancy of ≤6 months at baseline, but the actual time from enrolment to death varied from ~1–6 months across participants; the 3 monthly assessments immediately preceding death were therefore the only time points at which all participants had been assessed at the same standardised intervals from a common anchor (the death). This common anchoring was required for valid trajectory modelling across participants. Second, anticipatory grief is conceptually most relevant in the period immediately preceding the death; restricting the trajectory window to the final 3 months focused the analysis on this clinically meaningful interval, and post‐death grief trajectories were examined at 0, 3 and 6 months after bereavement. A second‐order polynomial function was applied, consistent with previous studies of grief trajectories [10, 19]. The optimal number of trajectory subgroups (1–6) was determined based on the following criteria: (1) the Bayesian information criterion (BIC), with lower absolute values indicating a superior model fit; (2) an average posterior probability (AvePP, defined as the mean of the model‐estimated probabilities of group membership for participants assigned to a given group, with values closer to 1 indicating higher classification certainty) > 0.70; and a minimum class size of no less than 2% of the population [20]. The final model was selected based on theoretical clarity and adequate subgroup sample sizes. Initially, a per‐protocol sensitivity analysis was planned using GBTM by excluding participants with LES scores ≥20 to focus our findings solely on bereavement; however, no such cases were reported during the follow‐up period. A *p* < 0.05 was considered statistically significant. All statistical analyses were performed with IBM SPSS Statistics (Version 29.0.1.0 [171]) and R software (Version 2022.12.0 + 353).

## 3. Results

### 3.1. Participants

A total of 86 immediate family members of terminally ill patients were screened between October and December 2023. The first participant was recruited on 20 October, 2023. Of these, 26 did not meet the inclusion criteria. The study enrolled 60 participants with a mean (SD) age of 52.82 (25–74 years; 10.18%); 32 (53.3%) were women, and 32 (53.3%) reported a history of physical illness. Most participants were offspring of terminally ill patients (44 [73.3%]), followed by spouses (6 [10.0%]). The mean (SD) score on the VAS‐Closeness Scale was 6.82 (1.68). The median (IQR) estimated life expectancy of terminally ill patients, based on the medical judgment, was 5 (4–6) months. Table 1 presents the demographic and clinical characteristics of the participants.

**Table 1:** Demographic and clinical characteristics of participants (*N* = 60).

Characteristics	*n* (%), mean ± SD/median (Q1, Q3)
Sex	
Female	32 (53.3%)
Male	28 (46.7%)
Age (years)	52.82 ± 10.18
20–40	5 (8.3%)
41–60	42 (70.0%)
61–80	13 (21.7%)
Ethnicity (Han)	60 (100.0%)
Education level	
Illiterate	0 (0%)
Primary school	7 (11.7%)
Middle school	12 (20.0%)
High school/vocational school	15 (25.0%)
Associate degree	15 (25.0%)
University and above	11 (18.3%)
Residence	
Rural	60 (100.0%)
Urban	0 (0%)
Monthly household income (CNY)	
1000–2000	13 (21.7%)
2000–5000	22 (36.7%)
5000–10,000	25 (41.7%)
Dying being the primary financial provider	
No	33 (55.0%)
Yes	27 (45.0%)
Marital status	
Unmarried	3 (5.0%)
Married	54 (90.0%)
Divorced	2 (3.3%)
Widowed	1 (1.7%)
Drinking history	
No	43 (71.7%)
Yes	17 (28.3%)
Smoking history	
No	31 (51.7%)
Yes	29 (48.3%)
Substance abuse history (no)	60 (100.0%)
Family history of psychiatric illness (no)	0 (0%)
Relationship with terminally ill patient	
Spouse	6 (10.0%)
Offspring	44 (73.3%)
Other (foster parent/child)	10 (16.7%)
Primary diagnosis of terminally ill patient	
Respiratory system	9 (15.0%)
Digestive system	2 (3.3%)
Cardiovascular system	11 (18.3%)
Cerebrovascular system	20 (33.3%)
Nervous system	14 (23.3%)
Urogenital system	1 (1.7%)
Other	3 (5.0%)
History of physical illness	
None	28 (46.7%)
Yes	32 (53.3%)
Estimated life expectancy, median (IQR) (months)	5 (4, 6)
Number of past physical illnesses, median (IQR)	1 (0, 1)
VAS‐Closeness Score, mean (SD)^a^	6.82 ± 1.68

^a^VAS‐Closeness Scale: range, 0–10. A score of 0 indicates ‘I have minimal emotional connection with the family member who is about to pass away. His/her departure has no impact on me. Compared to the love I have for my most beloved person, my love for him/her is almost negligible’; 10, ‘I have a deep emotional connection with the family member who is about to pass away. I cannot imagine how I would cope without him/her. Compared to the love I have for my most beloved person, my love for thim/her is equal to or even greater’.

### 3.2. Changes in AGS and PG‐13‐R, HAMD‐17, HAMA and IES‐R Total Score

Pre‐death AGS scores increased over time, whereas post‐death PG‐13‐R scores decreased. Similarly, pre‐death HAMD‐17, HAMA and IES‐R scores increased, while these scores decreased during the post‐death period. Figure 1 illustrates these trends.

**Figure 1:**
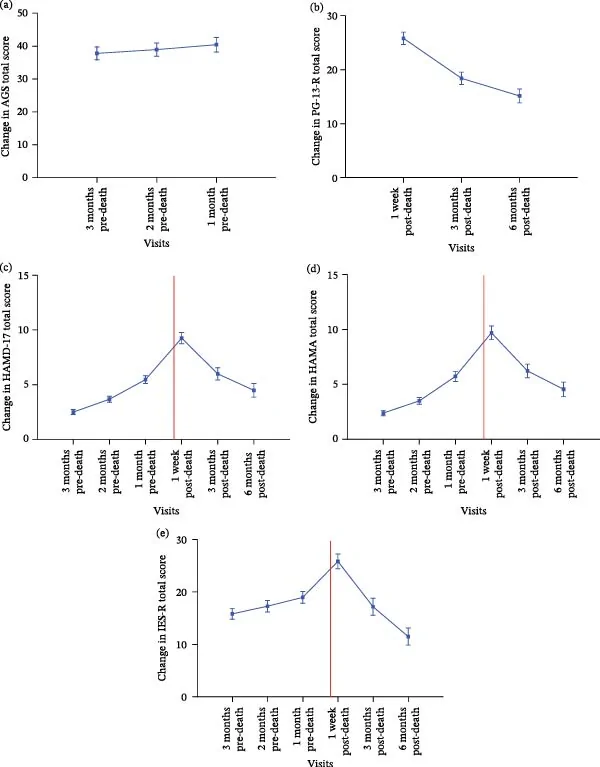
Change in AGS, PG‐13‐R, HAMD‐17, HAMA and IES‐R total score. (a) Change in AGS total score; AGS, the Anticipatory Grief Scale (range, 27–135, with higher score indicating higher level of anticipatory grief); (b) Change in PG‐13‐R total score; PG‐13‐R, the Prolonged Grief Disorder‐13 Revised (range, 10–50, with higher score indicating higher level of post‐death grief); (c) Change in HAMD‐17 total score; HAMD‐17, the 17‐item Hamilton Rating Scale for Depression (range, 0–52, with higher score indicating more severe depressive symptoms); (d) Change in HAMA total score; HAMA, the Hamilton Anxiety Scale (range, 0–56, with higher score indicating more severe anxiety symptoms); (e) Change in IES‐R total score; IES‐R, the Impact of Event Scale‐Revised (range 0–88, with higher score indicating more severe stress symptoms). Error bars indicate SEs.

### 3.3. Trajectories of Grief, Depression, Anxiety and Stress

We used GBTM to fit models of 1–6 groups to the pre‐death AGS scores. The two‐group model exhibited the lowest BIC value (1151.78). These two groups were classified as a low pre‐death grief group (*n* = 51 [85%]) and an increasing pre‐death grief group (*n* = 9 [15%]). The AvePPs for these groups were 0.99 and 0.93, respectively. Similarly, when modelling PG‐13‐R scores at multiple post‐death time points (1–6 groups), the optimal fit was a four‐group model (BIC = 1060.69). The four post‐death groups identified were high/decreasing (*n* = 9 [15%]), moderate/decreasing (*n* = 26 [43.33%]), low/decreasing (*n* = 15 [25%]) and high/prolonged (*n* = 10 [16.67%]). The AvePP values for these four groups were 0.93, 0.95, 0.98 and 0.93, respectively. Additionally, we applied GBTM to HAMD‐17, HAMA and IES‐R scores at pre‐ and post‐death visits. Among pre‐death trajectories, we identified two distinct trajectories of depression and stress and three distinct patterns of anxiety. Post‐death trajectories included three distinct trajectories of anxiety and depression and four distinct patterns of stress. These findings are illustrated in Figure 2.

Figure 2Longitudinal trajectories of grief, depression, anxiety and stress. AGS, the Anticipatory Grief Scale (range, 27–135, with higher score indicating higher level of anticipatory grief); PG‐13‐R, the Prolonged Grief Disorder‐13 Revised (range, 10–50, with higher score indicating higher level of post‐death grief); HAMD‐17, the 17‐item Hamilton Rating Scale for Depression (range, 0–52, with higher score indicating more severe depressive symptoms); HAMA, the Hamilton Anxiety Scale (range, 0–56, with higher score indicating more severe anxiety symptoms); IES‐R, the Impact of Event Scale‐Revised (range 0–88, with higher score indicating more severe stress symptoms); BIC, Bayesian information criterion; AIC, Akaike information criterion; AvePP, average posterior probability. (a) Pre‐death grief; class 1: low; class 2: increasing. (b) Post‐death grief; class 1: high/decreasing; class 2: moderate/decreasing; class 3: low/decreasing; class 4: high/prolonged. (c) Pre‐death depression; class 1: slowly increasing; class 2: rapidly increasing. (d) Post‐death depression; class 1: high/decreasing; class 2: low/decreasing; class 3: high. (e) Pre‐death anxiety; class 1: low/increasing; class 2: moderate/increasing; class 3: high/increasing. (f) Post‐death anxiety; class 1: high/rapidly decreasing; class 2: low/slowly decreasing; class 3: high/slowly decreasing. (g) Pre‐death stress; class 1: persistently low; class 2: high/increasing. (h) Post‐death stress; class 1: low/decreasing; class 2: moderate/decreasing; class 3: high/decreasing; class 4: high.

**Figure:**
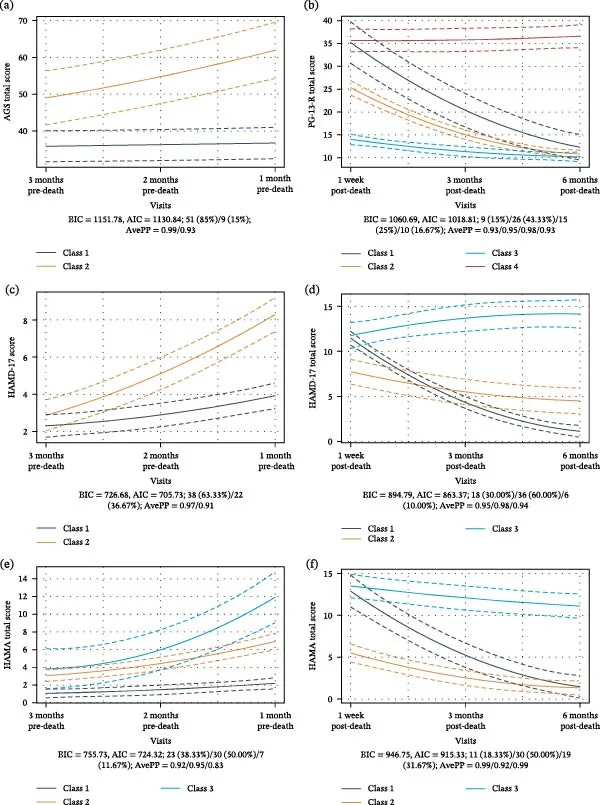

**Figure:**
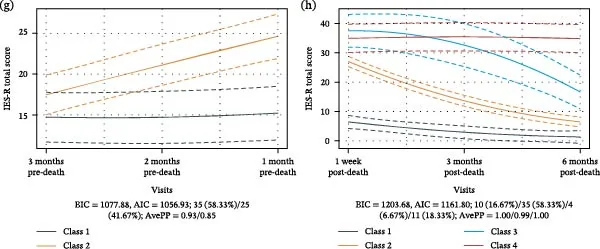

The pre‐death grief trajectory was correlated with the post‐death grief trajectory (Cramer’s *V* = 0.695, *p* < 0.001). Because pre‐death and post‐death grief trajectories were derived from non‐overlapping symptom scores (AGS during the pre‐death period and PG‐13‐R during the post‐death period), this association reflects a substantive longitudinal relationship between the two grief constructs rather than a methodological artefact arising from shared input variables. Neither grief trajectory was correlated with the trajectories of depression, anxiety or stress (Figure S1).

### 3.4. Comparison of Demographic and Clinical Characteristics Across Trajectory Groups

Participants in the low grief group had a higher medically estimated remaining life expectancy (mean [SD], 4.98 [1.10] vs. 3.44 [1.42]; *p* < 0.001) and lower VAS‐Closeness Scale scores (mean [SD], 6.57 [1.65] vs. 8.22 [1.09]; *p* = 0.006) than the increasing grief group (Table 2). There were also substantial differences between groups in the primary diagnostic system classification of the terminally ill patient’s condition (*χ* ^2^ = 13.916, *p* = 0.031), with cerebrovascular conditions most common in the low grief group (35.3%), while respiratory conditions were most common in the increasing grief group (33.3%). LES scores did not differ substantially between trajectory groups, indicating that the observed trajectory differences were not attributable to differences in concurrent positive or negative life events.

**Table 2:** Demographic and clinical characteristics across specific grief trajectories (*N* = 60).

Characteristics	Participants, number (%)					
	Low pre‐death grief (*n* = 51)	Increasing pre‐death grief (*n* = 9)	High/decreasing post‐death grief (*n* = 9)	Moderate/decreasing post‐deathgrief (*n* = 26)	Low/decreasing post‐death grief (*n* = 15)	High/prolonged post‐death grief (*n* = 10)
Sex						
Female	27 (52.90)	5 (55.60)	4 (44.4)	14 (53.8)	7 (46.7)	7 (70.0)
Male	24 (47.10)	4 (44.40)	5 (55.6)	12 (46.2)	8 (53.3)	3 (30.0)
Age (years)						
20–40	4 (7.8)	1 (11.1)	1 (11.1)	1 (3.8)	2 (13.3)	1 (10.0)
41–60	37 (72.5)	5 (55.6)	4 (44.4)	21 (80.8)	9 (60.0)	8 (80.0)
61–80	10 (19.6)	3 (33.3)	4 (44.4)	4 (15.4)	4 (26.7)	1 (10.0)
Ethnicity (Han)	51 (100)	9 (100)	9 (100)	26 (100)	15 (100)	10 (100)
Education level						
Illiterate	0 (0)	0 (0)	0 (0)	0 (0)	0 (0)	0 (0)
Primary school	5 (9.8)	2 (22.2)	2 (22.2)	1 (3.8)	1 (6.7)	3 (30.0)
Middle school	9 (17.6)	3 (33.3)	0 (0)	5 (19.2)	3 (20.0)	4 (40.0)
High school/vocational school	14 (27.5)	1 (11.1)	3 (33.3)	10 (38.5)	1 (6.7)	1 (10.0)
Associate degree	13 (25.5)	2 (22.2)	2 (22.2)	8 (30.8)	4 (26.7)	1 (10.0)
University and above	10 (19.6)	1 (11.1)	2 (22.2)	2 (7.7)	6 (40.0)	1 (10.0)
Residence						
Rural	0 (0)	0 (0)	0 (0)	0 (0)	0 (0)	0 (0)
Urban	51 (100)	9 (100)	9 (100)	26 (100)	15 (100)	10 (100)
Monthly household income (CNY)						
1000–2000	10 (19.6)	3 (33.3)	3 (33.3)	5 (19.2)	1 (6.7)	4 (40.0)
2000–5000	19 (37.3)	3 (33.3)	4 (44.4)	8 (30.8)	7 (46.7)	3 (30.0)
5000–10,000	22 (43.1)	3 (33.3)	2 (22.2)	13 (50.0)	7 (46.7)	3 (30.0)
Dying being the primary financial provider						
No	30 (58.8)	3 (33.3)	6 (66.7)	13 (50.0)	11 (73.3)	3 (30.0)
Yes	21 (41.2)	6 (66.7)	3 (33.3)	13 (50.0)	4 (26.7)	7 (70.0)
Marital status						
Unmarried	3 (5.9)	0 (0)	1 (11.1)	0 (0)	0 (0)	0 (0)
Married	46 (90.2)	8 (88.9)	8 (88.9)	25 (96.2)	2 (13.3)	9 (90.0)
Divorced	2 (3.9)	0 (0)	0 (0)	1 (3.8)	12 (80.0)	0 (0)
Widowed	0 (0)	1 (11.1)	0 (0)	0 (0)	1 (6.7)	1 (10.0)
Drinking history						
No	38 (74.5)	5 (55.6)	7 (77.8)	16 (61.5)	13 (86.7)	7 (70.0)
Yes	13 (25.5)	4 (44.4)	2 (22.2)	10 (38.5)	2 (13.3)	3 (30.0)
Smoking history						
No	26 (51.0)	5 (55.6)	5 (55.6)	13 (50.0)	6 (40.0)	7 (70.0)
Yes	25 (49.0)	4 (44.4)	4 (44.4)	13 (50.0)	9 (60.0)	3 (30.0)
Substance abuse history (no)	51 (100.0)	9 (100.0)	9 (100.0)	26 (100.0)	15 (100.0)	10 (100.0)
Family history of psychiatric illness (no)	0 (0)	0 (0)	9 (100.0)	26 (100.0)	15 (100.0)	10 (100.0)
Relationship with terminally ill patient						
Spouse	6 (11.8)	0 (0)	2 (22.2)	1 (3.8)	2 (13.3)	1 (10.0)
Offspring	38 (74.5)	6 (66.7)	7 (77.8)	25 (96.2)	6 (40.0)	6 (60.0)
Other (foster parent/child)	7 (13.7)	3 (33.3)	0 (0)	0 (0)	7 (46.7)	3 (30.0)
Primary diagnosis of terminally ill patient						
Respiratory system	6 (11.8)	3 (33.3)	1 (11.1)	3 (11.5)	1 (6.7)	4 (40.0)
Digestive system	1 (2.0)	1 (11.1)	0 (0)	0 (0)	1 (6.7)	1 (10.0)
Cardiovascular system	10 (19.6)	1 (11.1)	0 (0)	2 (7.7)	6 (40.0)	3 (30.0)
Cerebrovascular system	18 (35.3)	2 (22.2)	5 (55.6)	11 (42.3)	4 (26.7)	0 (0)
Nervous system	14 (27.5)	0 (0)	2 (22.2)	9 (34.6)	3 (20.0)	0 (0)
Urogenital system	0 (0)	1 (11.1)	0 (0)	0 (0)	0 (0)	1 (10.0)
Other	2 (3.9)	1 (11.1)	1 (11.1)	1 (3.8)	0 (0)	1 (10.0)
History of physical illness						
None	23 (45.1)	5 (55.6)	3 (33.3)	15 (57.7)	7 (46.7)	3 (30.0)
Yes	28 (54.9)	4 (44.4)	6 (66.7)	11 (42.3)	8 (53.3)	7 (70.0)
Estimated life expectancy, median (IQR) (months)	5.00 (4.00, 6.00)	3.00 (2.00, 4.50)	5.00 (3.50, 5.00)	5.50 (5.00, 6.00)	5.00 (5.00, 6.00)	3.00 (2.00, 3.25)
Number of past physical illnesses, median (IQR)	1.00 (0.00, 1.00)	0.00 (0.00, 1.00)	1.00 (0.00, 1.00)	0.00 (0.00, 1.00)	1.00 (0.00, 1.00)	1.00 (0.00, 2.25)
VAS‐Closeness Score, mean (SD)^a^	6.57 ± 1.65	8.22 ± 1.09	7.00 ± 0.87	7.12 ± 0.86	4.87 ± 1.51	8.80 ± 1.14

^a^VAS‐Closeness Scale: range, 0–10. A score of 0 indicates ‘I have minimal emotional connection with the family member who is about to pass away. His/her departure has no impact on me. Compared to the love I have for my most beloved person, my love for him/her is almost negligible’; 10, ‘I have a deep emotional connection with the family member who is about to pass away. I cannot imagine how I would cope without him/her. Compared to the love I have for my most beloved person, my love for thim/her is equal to or even greater’.

Among the four post‐death grief trajectory groups, substantial differences were observed in education level (*χ* ^2^ = 21.474, *p* = 0.044), relationship to the terminally ill patient (*χ* ^2^ = 21.882, *p* = 0.001), primary diagnostic system classification (*χ* ^2^ = 32.313, *p* = 0.020), medically estimated remaining life expectancy (*Z* = 27.039, *p* < 0.001), and VAS‐Closeness Scale scores (*F*_(3,56)_ = 27.181, *p* < 0.001). Participants in the high/prolonged grief group had the lowest medically estimated remaining life expectancy (mean [SD], 3.00 [2.00–3.25] months) and the highest VAS‐Closeness Scale scores (mean [SD], 8.80 [1.14]) than the other groups. The low/decreasing group had the highest proportion of participants with a university degree or above (40.0%), whereas most participants in the high/prolonged grief group had a middle school education (40.0%). The low/decreasing group had the highest proportion of participants classified as ‘other’ (46.7%), whereas offspring were the most common in the other groups. Cardiovascular disorders were most common in the low/decreasing group (40.0%), while respiratory disorders were most common in the high/prolonged group (40.0%). As mentioned above, all comparisons across trajectory groups used variables external to the GBTM models that derived the trajectories.

No substantial differences in demographic and clinical characteristics were observed across the depression (Table S1), anxiety (Table S2), and stress trajectories (Table S3).

### 3.5. Incidences of PGD and Other Mental Disorders at 6 Months Post‐Bereavement

The incidences of PGD, depressive disorders, anxiety disorders, and stress‐related disorders were 16.7%, 16.7%, 30.0% and 0%, respectively, at the end of this study. In the increasing pre‐death grief group, the incidences of PGD, MDD and GAD were 77.8%, 55.6% and 100.0%, respectively, which were significantly higher than those in the low pre‐death grief group (5.9%, 9.8% and 17.6%, respectively; *χ* ^2^ = 28.471, *p* < 0.001 for PGD; *χ* ^2^ = 11.529, *p* < 00.001 for MDD; and *χ* ^2^ = 24.706, *p* < 0.001 for GAD). In the high/prolonged post‐death grief group, the incidences of PGD, MDD and GAD were 100.0%, 60.0% and 100.0%, respectively, which were significantly higher than those in the other decreasing trajectory groups (*χ* ^2^ = 60.000, *p* < 0.001 for PGD; *χ* ^2^ = 21.397, *p* < 0.001 for MDD; and *χ* ^2^ = 29.915, *p* < 0.001 for GAD). However, no significant differences were observed in the incidences of PGD, depressive disorders, anxiety disorders or stress‐related disorders among the various pre‐ and post‐death depression, anxiety or stress trajectories (Table 3).

**Table 3:** Incidences of PGD, MDD, GAD and stress‐related disorders across trajectories.

Identified trajectories	PGD, *n* (%)	MDD, *n* (%)	GAD, *n* (%)	Stress‐related disorders, *n* (%)
Pre‐death grief trajectories				
Low pre‐death grief (*n* = 51)	3 (5.9)	5 (9.8)	9 (17.6)	0 (0)
Increasing pre‐death grief (*n* = 9)	7 (77.8)	5 (55.6)	9 (100.0)	0 (0)
*χ* ^2^	28.471	11.529	24.706	—
*p*	**< 0.001**	**< 0.001**	**< 0.001**	—
Post‐death grief trajectories				
High/decreasing post‐death grief (*n* = 9)	0 (0)	3 (33.3)	3 (33.3)	0 (0)
Moderate/decreasing post‐death grief (*n* = 26)	0 (0)	1 (3.8)	4 (15.4)	0 (0)
Low/decreasing post‐death grief (*n* = 15)	0 (0)	0 (0)	1 (6.7)	0 (0)
High/prolonged post‐death grief (*n* = 10)	10 (100.0)	6 (60.0)	10 (100.0)	0 (0)
*χ* ^2^	60.000	21.397	29.915	—
*p*	**< 0.001**	**< 0.001**	**< 0.001**	—
Pre‐death depression trajectories				
Slowly increasing pre‐death depression (*n* = 38)	5 (13.2)	7 (18.4)	10 (26.3)	0 (0)
Rapidly increasing pre‐death depression (*n* = 22)	5 (22.7)	3 (13.6)	8 (36.4)	0 (0)
*χ* ^2^	0.919	0.230	0.670	—
*p*	0.338	0.632	0.413	—
Post‐death depression trajectories				
High‐level, continuously decreasing post‐death depression (*n* = 18)	3 (16.7)	3 (16.7)	4 (22.2)	0 (0)
Low‐level, continuously decreasing post‐death depression (*n* = 36)	7 (19.4)	7 (19.4)	13 (36.1)	0 (0)
Persistently high‐level post‐death depression (*n* = 6)	0 (0)	0 (0)	1 (16.7)	0 (0)
*χ* ^2^	1.400	1.400	1.667	—
*p*	0.497	0.497	0.435	—
Pre‐death anxiety trajectories				
Low‐level, continuously increasing pre‐death anxiety (*n* = 23)	4 (17.4)	6 (26.1)	7 (100.0)	0 (0)
Moderate‐level, continuously increasing pre‐death anxiety (*n* = 30)	5 (16.7)	3 (10.0)	30 (100.0)	0 (0)
High‐level, rapidly increasing pre‐death anxiety (*n* = 7)	1 (14.3)	1 (14.3)	7 (100.0)	0 (0)
*χ* ^2^	0.037	2.458	0.009	—
*p*	0.982	0.293	0.996	—
Post‐death anxiety trajectories				
High‐level, rapidly decreasing post‐death anxiety (*n* = 11)	1 (9.1)	2 (18.2)	2 (18.2)	0 (0)
Low‐level, continuously decreasing post‐death anxiety (*n* = 30)	6 (20.0)	5 (16.7)	9 (30.0)	0 (0)
High‐level, continuously decreasing post‐death anxiety (*n* = 19)	3 (15.8)	3 (15.8)	7 (36.8)	0 (0)
*χ* ^2^	0.705	0.029	1.155	—
*p*	0.703	0.986	0.561	—
Pre‐death stress trajectories				
Persistently low‐level pre‐death stress (*n* = 35)	7 (20.0)	7 (20.0)	11 (31.4)	0 (0)
High‐level continuously increasing pre‐death stress (*n* = 25)	3 (12.0)	3 (12.0)	7 (28.0)	0 (0)
*χ* ^2^	0.672	0.672	0.082	—
*p*	0.412	0.412	0.775	—
Post‐death stress trajectories				
Low‐level, continuously decreasing post‐death stress (*n* = 10)	1 (10.0)	1 (10.0)	3 (30.0)	0 (0)
Moderate‐level rapidly decreasing post‐death stress (*n* = 35)	7 (20.0)	6 (17.1)	9 (25.7)	0 (0)
High‐level rapidly decreasing post‐death stress (*n* = 4)	0 (0)	1 (25.0)	1 (25.0)	0 (0)
Persistently high‐level post‐death stress (*n* = 11)	2 (18.2)	2 (18.2)	5 (45.5)	0 (0)
*χ* ^2^	1.418	0.544	1.605	—
*p*	0.701	0.909	0.658	—

*Note:* *p* < 0.001 are shown in bold.

Abbreviations: GAD, generalised anxiety disorder; MDD, major depressive disorder; PGD, prolonged grief disorder.

## 4. Discussion

In this study, we examined the dynamic changes in grief, depression, anxiety and stress among immediate family members of terminally ill patients during the pre‐ and post‐death periods. These psychiatric symptoms gradually increased before bereavement and subsequently decreased afterward. We identified two distinct pre‐death grief trajectories (low and increasing) and four post‐death grief trajectories (high/decreasing, moderate/decreasing, low/decreasing and high/prolonged). Our findings indicate that most participants experienced persistently low levels of grief before bereavement, while a moderate level of grief gradually decreased after the loss. These observations address question (a) and provide preliminary support for the trajectory structure proposed in the PGD developmental atlas in a Chinese palliative care cohort. As an exploratory pilot study, the present findings should be regarded as candidate descriptive patterns to be refined in larger replication studies rather than as definitive characterisations of the trajectory landscape.

Generally, our findings were consistent with those of previous studies. Lundorff et al. identified four post‐death grief trajectories: resilient (64.4%; persistently low grief), moderate‐Stable (20.4%; persistently moderate grief), recovery (8.4%; initially high grief that gradually improved), and prolonged (6.8%; persistently high grief) [10]. Notably, the moderate‐stable group in Lundorff et al.’s study does not align precisely with the moderate/decreasing group identified in our study. The primary distinction is that although both groups exhibited moderate levels of grief at baseline, the moderate/decreasing group in our study showed a gradual reduction over time rather than remaining stable. The other three trajectories identified by Lundorff et al. can be roughly matched with those observed in our study (resilient vs. low/decreasing, recovery vs. high/decreasing, and prolonged vs. high/prolonged). Another study supports these findings [21]. The observed differences in trajectory characteristics may be due to several factors, including the smaller sample size in our study, which may limit the ability to fully characterise the stable progression of grief trajectories. Cultural factors may also contribute to the differences in trajectory composition. Most prior longitudinal studies of post‐death grief trajectories have been conducted in Western populations, predominantly involving partner‐bereavement following spousal death, whereas the present cohort comprised mainly offspring of terminally ill parents in a Chinese cultural context. Chinese mourning practices, which emphasise filial piety, structured family rituals, and ongoing symbolic bonds with the deceased, may shape both the expression and the temporal course of grief in ways that differ from those documented in Western partner‐bereaved samples. Additionally, previous studies mostly focused on a single symptom (i.e., grief) after bereavement, with limited studies expanding their focus on various key psychiatric symptoms potentially relevant. A prospective study in which participants bereaved for less than 1 year completed follow‐up questionnaires every 6 weeks, up to a total of 11 assessments over 15 months [12]. Their findings revealed three distinct PG trajectories: resilient (66.4%), chronic (25.1%) and acute recovery (8.4%) and found that chronic stress in the first year was often associated with chronic grief and depression. However, our findings provide evidence that grief follows unique developmental pathways distinct from other psychiatric symptoms, such as depression, anxiety and stress. Specifically, we identified that the longitudinal trajectories of these symptoms vary and that pre‐death grief trajectories were substantially associated with post‐death grief trajectories but not with trajectories of other psychiatric symptoms. As noted in Section 2.5, this association reflects a relationship between latent groups derived from non‐overlapping variable sets (AGS for pre‐death; and PG‐13‐R for post‐death) rather than a methodological artefact, supporting question (b). These findings shed new light on the relative independence of grief as a distinct psychiatric construct with its developmental trajectory, reinforcing the idea that grief symptoms are autonomous phenomena rather than mere correlates of broader psychiatric distress.

Our findings demonstrated that pre‐ and post‐death grief responses were associated with both the characteristics of the terminally ill patient’s condition and the emotional dynamics of the caregiver. Immediate families of patients with shorter estimated life expectancies and more chronic and debilitating conditions (e.g., respiratory conditions, in this study) were more likely to experience a high or prolonged grief trajectory. This pattern is consistent with adult bereavement literature on the role of the manner and circumstances of death: prior work has shown that witnessing prolonged respiratory distress or other distressing terminal symptoms can intensify the emotional burden on family members and amplify the risk of complicated grief [15], whereas cerebrovascular conditions, often involving sudden incapacitation rather than a protracted dying process, may impose a different, though not necessarily lesser, grief burden. We emphasise, however, that these patterns emerged in a small, single‐centre cohort and that the cerebrovascular vs. respiratory contrast should be regarded as hypothesis‐generating. Replication in larger cohorts that systematically capture circumstances‐of‐death variables is needed before causal or theoretical inferences can be drawn. A mixed‐methods study by Moore et al. found that emotion‐oriented coping strategies were associated with lower grief intensity, while dysfunctional coping strategies correlated with higher grief levels, suggesting that caregivers benefit from psychological strategies such as acceptance and humour, as well as seeking support [15]. A longer anticipated bereavement period may allow for more time to prepare emotionally and engage in coping strategies. Additionally, greater emotional attachment was also associated with high or increasing grief.

In this study, 16.7% of participants developed PGD, which was slightly higher than the PGD prevalence reported in previous studies. A meta‐analysis estimated a pooled prevalence of PGD at 9.8% in non‐psychiatric adult populations exposed to non‐violent bereavement but lower than the ~50% prevalence reported among bereaved individuals following unnatural losses [6, 7]. The incidence of MDD was 30.0%, consistent with the findings of previous studies. However, unlike previous studies that observed PTSD rates as high as 33.9%, no participants in this study were diagnosed with PTSD [14]. Several factors may contribute to the absence of PTSD cases in our cohort. Unlike sudden or unexpected losses, the terminal illness and anticipated death of patients may have allowed family members to psychologically prepare for bereavement, mitigating the traumatic impact commonly observed in cases of unexpected loss. Additionally, the relatively small sample size of this pilot cohort may have limited the ability to detect less common disorders. Diagnostic confidence in the present cohort was supported by the use of two‐rater clinical diagnostic interviews with ICD‐11 criteria and confirmation only on the independent agreement of both psychiatrists; nevertheless, the ICD‐11 PGD criteria and the related ICD‐11 categories were originally developed and validated principally in Western and mixed international samples, and their full cultural calibration in Chinese palliative‐care populations is still an open question that warrants dedicated future work. Aside from significant differences in the incidences of PGD, depressive disorders and anxiety disorders between pre‐death and post‐death grief trajectories, no significant differences were observed between depression, anxiety and stress trajectories. These findings suggest that more attention should be directed toward individuals who experience increasing pre‐death grief or high/prolonged post‐death grief to prevent adverse mental health outcomes. Within the constraints of an exploratory design, the candidate correlates identified here, short estimated remaining life expectancy, respiratory terminal illness, lower educational attainment and higher emotional closeness, may be most usefully understood as risk markers warranting prospective testing rather than as causal determinants. Factors such as the chronicity and debilitation of the terminally ill patient’s condition and greater emotional closeness may help identify these vulnerable populations. Preventive interventions, including ultra‐early intervention and early intervention for pathological grief, may involve low‐intensity psychotherapy tailored to the heterogeneity of grief experiences and individual’s medical care needs rather than employing one‐size‐fits‐all protocols [8].

### 4.1. Strengths, Limitations and Directions for Future Research

This pilot study has several strengths. To our knowledge, it is among the first prospective cohort studies of grief in a Chinese palliative care setting to span both the pre‐death and post‐death periods within the same participants. The use of standardised clinician‐rated and self‐report measures across both periods, monthly pre‐death and quarterly post‐death assessments, and GBTM on non‐overlapping variable sets supports the interpretability of the findings. This study has some limitations. First, given the novelty of conducting a prospective longitudinal study spanning both pre‐ and post‐death periods, significant resources were allocated to assessing the cultural acceptability and feasibility. As a result, we adopted a pilot study design, which limited the sample size, thereby restricting the robustness and generalisability of our findings. The small sample size limited our ability to conduct detailed subgroup analyses, including investigating differences based on the disease classifications of terminally ill patients. Besides, it constrained our capacity to develop robust regression models for identifying protective and risk factors associated with high‐risk grief trajectories. Furthermore, the relatively small sample size may not have accurately represented the spectrum of mental disorders or their true incidences among bereaved populations. For instance, the absence of PTSD cases in this study may indicate a false‐negative result due to insufficient statistical power. Second, the dimensional symptom measures used as inputs to trajectory modelling (AGS, PG‐13‐R, HAMD‐17, HAMA and IES‐R) are self‐report or interview‐rated scales validated primarily in adult clinical and community populations; reliance on these instruments for the trajectory inputs introduces potential biases, including social‐desirability effects, recall bias and the possibility that culturally specific expressions of distress are underdetected by Western‐derived measures. Diagnostic outcomes themselves were established by an independent two‐rater clinical diagnostic interview using ICD‐11 criteria, which provides stronger diagnostic specificity than scale‐based caseness; nevertheless, the ICD‐11 PGD criteria were originally developed and validated principally in Western and mixed international samples and their full cultural calibration in Chinese palliative‐care populations remains an open question. Third, the post‐death follow‐up period was limited to 6 months and PGD diagnoses were made exclusively based on the ICD‐11 criteria. This method restricted the ability to assess PGD using DSM‐5‐TR criteria, limiting the capacity of the study to provide comprehensive data supporting the diagnostic frameworks. Fourth, the nature and context of bereavement may differentially influence grief responses. Since our study focused exclusively on immediate family members of terminally ill patients, it may not fully capture the broader psychiatric phenomena associated with other types of bereavement, including sudden loss or violent death. Fifth, although the PG‐13‐R includes an item assessing functional impairment and the ICD‐11 diagnostic interviews also considered functional impairment, the present design did not include a measure of functional impairment, overall psychological well‐being or resilient recovery; symptom‐based outcomes alone cannot capture positive adjustment, role functioning or quality of life, all of which are increasingly emphasised in contemporary bereavement research. Finally, this is an unreplicated single‐centre pilot, and trajectory solutions derived in small samples are known to be susceptible to chance variation. In future studies, we will address these limitations by expanding sample sizes, extending follow‐up periods, using dual diagnostic frameworks (ICD‐11 and DSM‐5‐TR) and including diverse bereavement contexts to provide a more comprehensive understanding of grief trajectories and associated mental health outcomes. In light of these limitations, future research should (1) replicate and cross‐validate the trajectory solutions identified here in a larger, ideally multicentre, cohort with sufficient power to model trajectories within and across diagnostic and demographic subgroups; (2) incorporate measures of functional impairment, psychological well‐being and resilient recovery alongside symptom severity, so that predicted outcomes do not overlap heavily with predictors of trajectory membership; (3) systematically capture candidate vulnerability and protective factors (e.g., attachment style, prior trauma exposure, social support and coping repertoire) and potential mediators (e.g., loss reminders and secondary stressors) within an explicit theoretical framework such as the PGD developmental atlas; (4) employ designs that better support causal inference, including the use of pre‐specified mediation and moderation analyses and theoretically motivated longitudinal modelling; and (5) align findings with their methodologically supported applications, including correlates of trajectory membership as candidate risk markers, predictors as candidate prognostic indicators and identification of time windows in which prognosis becomes apparent and which may be used to flag individuals at elevated risk for sustained distress.

## 5. Conclusion

Individuals who experienced increasing pre‐death grief or persistently high‐level post‐death grief were more likely to develop PGD and other adverse mental health outcomes. Factors such as lower education level, the chronicity and debilitation of the terminally ill patient’s condition and greater emotional closeness may help identify these vulnerable populations; more attention is required to develop strategies for these vulnerable individuals. Within the limits of this exploratory pilot, these factors are best treated as candidate risk markers and prognostic indicators warranting cross‐validation in larger, theoretically informed cohorts before any specific intervention recommendations can be made.

## Author Contributions

Concept and design: Chunfeng Xiao and Jing Wei. Acquisition, analysis or interpretation of data: Chunfeng Xiao, Song Wan, Xinpeng Xu, Wentao Yao and Yufei Wang. Draughting of the manuscript: Chunfeng Xiao. Critical review of the manuscript for important intellectual content, obtained funding, administrative, technical or material support, supervision: Jing Wei. Statistical analysis: Chunfeng Xiao and Yufei Wang.

## Funding

This work was supported by research grants from the National Natural Science Foundation of China (Grant T2341003) and the Capital Fund for Health Improvement and Research (Grant 2022‐2‐4012).

## Disclosure

The funders had no role in the design and conduct of the study; collection, management, analysis and interpretation of the data; preparation, review or approval of the manuscript; and decision to submit the manuscript for publication. Jing Wei had full access to all data in the study and took responsibility for the integrity of the data and the accuracy of the data analysis.

## Conflicts of Interest

The authors declare no conflicts of interest.

## Supporting Information

Additional supporting information can be found online in the Supporting Information section.

## Supporting information


**Supporting Information** The following Supporting Information are available in Supplement 1 with the online version of this article. Additional figure and tables supporting the main analyses. Figure S1: Correlation coefficients between trajectories of grief, depression, anxiety and stress (pre‐death and post‐death), illustrating the associations among the latent trajectory groups described in Section 3. Table S1: Demographic and clinical characteristics stratified by depression trajectories (pre‐death and post‐death); values shown as *n* (%) or mean/SD or median/IQR as appropriate (*N* = 60). Table S2: Demographic and clinical characteristics stratified by anxiety trajectories (pre‐death and post‐death); values shown as *n* (%) or mean/SD or median/IQR as appropriate (*N* = 60). Table S3: Demographic and clinical characteristics stratified by stress trajectories (pre‐death and post‐death); values shown as *n* (%) or mean/SD or median/IQR as appropriate (*N* = 60).

## Data Availability

The datasets used and analysed during the current study are available from the corresponding author upon reasonable request.
