# Supplementary material for: Longitudinal Trajectories of Grief Before and After Bereavement and Their Association With Mental Health Outcomes: A Pilot Prospective Cohort Study From the China Grief Cohort 2025
Source: Depress Anxiety. 2026 Sep 28;2026:5274616. doi: 10.1155/da/5274616 (PMC13620408; doi:10.1155/da/5274616)
Supplement: Supplementary file 1 [file DA-2026-5274616-s001.pdf]

Fig. S1: Correlation coefficient between trajectories of grief, depression, anxiety, and stress

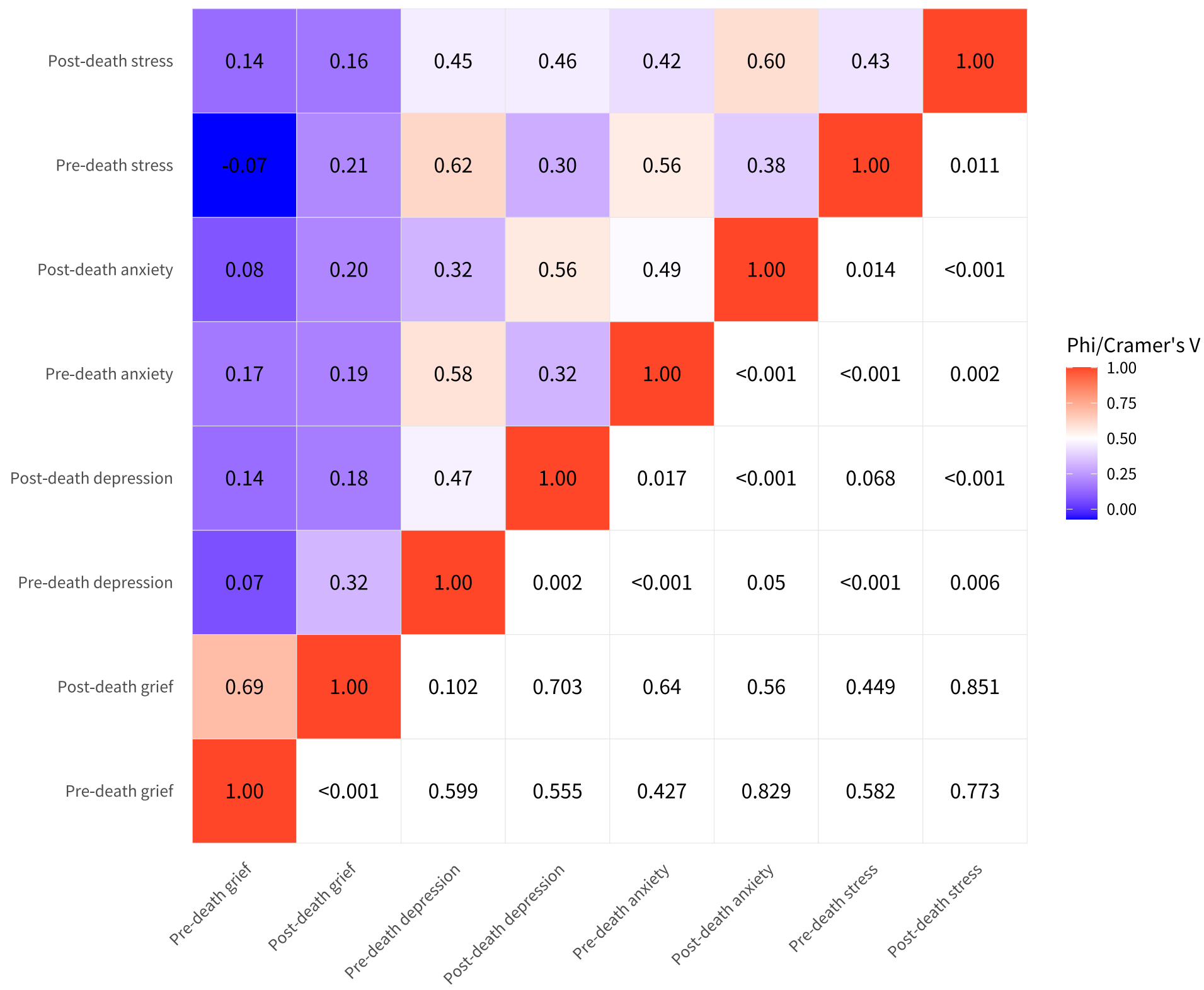

Tab. S1: Demographic and Clinical Characteristics Across Specific Depression Trajectories (N = 60).

Characteristics	Participants, No. (%)				
	Slowly increasing pre-death depression (n = 38)	Rapidly increasing pre-death depression (n = 22)	High-level, continuously decreasing post-death depression (n = 18)	Low-level, continuously decreasing post-death depression (n = 36)	Persistently high-level post-death depression (n = 6)
**Sex**					
Female	17 (44.74)	15 (68.18)	10 (55.6)	17 (47.2)	5 (83.3)
Male	21 (55.26)	7 (31.82)	8 (44.4)	19 (52.8)	1 (16.7)
**Age, y**	52.42±9.40	53.50±11.59	52.89±8.88	52.17±11.24	56.50±7.06
20–40	3 (7.9)	2 (9.1)	1 (5.6)	4 (11.1)	0 (0)
41–60	28 (73.7)	14 (63.6)	14 (77.8)	24 (66.7)	4 (66.7)
61–80	7 (18.4)	6 (27.3)	3 (16.7)	8 (22.2)	2 (33.3)
**Ethnicity (Han)**	38 (100)	22 (100)	18 (100)	36 (100)	6 (100)
**Education Level**					
Illiterate	0 (0)	0 (0)	0 (0)	0 (0)	0 (0)
Primary School	3 (9.8)	4 (18.2)	3 (16.7)	4 (11.1)	0 (0)
Middle School	7 (17.6)	5 (22.7)	3 (16.7)	7 (19.4)	2 (33.3)
High School/Vocational School	13 (27.5)	2 (9.1)	3 (16.7)	11 (30.6)	1 (16.7)
Associate Degree	10 (25.5)	5 (22.7)	6 (33.3)	8 (22.2)	1 (16.7)
University and Above	5 (19.6)	6 (27.3)	3 (16.7)	6 (16.7)	2 (33.3)
**Residence**					
Rural	0 (0)	0 (0)	0 (0)	0 (0)	0 (0)
Urban	38 (100)	22 (100)	18 (100)	36 (100)	6 (100)
**Monthly Household Income (CNY)**					
1,000–2,000	7 (18.4)	6 (27.3)	6 (33.3)	6 (16.7)	1 (16.7)
2,000–5,000	15 (39.5)	7 (31.8)	4 (22.2)	17 (47.2)	1 (16.7)
5,000–10,000	16 (42.1)	9 (40.9)	8 (44.4)	13 (36.1)	4 (66.7)
**Dying being the Primary Financial Provider**					
No	22 (57.9)	11 (50.0)	10 (55.6)	20 (55.6)	3 (50.0)
Yes	16 (42.1)	11 (50.0)	8 (44.4)	16 (44.4)	3 (50.0)
**Marital Status**					
Unmarried	1 (2.6)	2 (9.1)	1 (5.6)	2 (5.6)	0 (0)
Married	36 (94.7)	18 (81.8)	17 (94.4)	32 (88.9)	5 (83.3)
Divorced	1 (2.6)	1 (4.5)	0 (0)	1 (2.8)	1 (16.7)
Widowed	0 (0)	1 (4.5)	0 (0)	1 (2.8)	0 (0)
**Drinking History**					
No	26 (68.4)	17 (77.3)	13 (72.2)	25 (69.4)	5 (83.3)
Yes	12 (31.6)	5 (22.7)	5 (27.8)	11 (30.6)	1 (16.7)
**Smoking History**					
No	17 (44.7)	14 (63.6)	11 (61.1)	18 (50.0)	2 (33.3)

Yes	21 (55.2)	8 (36.4)	7 (38.9)	18 (50.0)	4 (66.7)
**Substance Abuse History (No)**	38 (100.0)	22 (100.0)	18 (100.0)	36 (100.0)	6 (100.0)
**Family History of Psychiatric Illness (No)**	38 (100.0)	22 (100.0)	18 (100.0)	36 (100.0)	6 (100.0)
**Relationship with Terminally Ill Patient**					
Spouse	2 (5.3)	4 (18.2)	3 (16.7)	2 (5.6)	1 (16.7)
Offspring	31 (81.6)	13 (59.1)	12 (66.7)	27 (75.0)	5 (83.3)
Other (foster parent/child)	5 (13.2)	5 (22.7)	3 (16.7)	7 (19.4)	0 (0)
**Primary Diagnosis of Terminally Ill Patient**					
Respiratory System	8 (21.1)	1 (4.5)	2 (11.1)	7 (19.4)	0 (0)
Digestive System	0 (0)	2 (9.1)	2 (11.1)	0 (0)	0 (0)
Cardiovascular System	5 (13.2)	6 (27.3)	4 (22.2)	6 (16.7)	1 (16.7)
Cerebrovascular System	12 (31.6)	8 (36.4)	5 (27.8)	12 (33.3)	3 (50.0)
Nervous System	11 (28.9)	3 (13.6)	4 (22.2)	8 (22.2)	2 (33.3)
Urogenital System	0 (0)	1 (4.5)	0 (0)	1 (2.8)	0 (0)
Other	2 (5.3)	1 (4.5)	1 (5.6)	2 (5.6)	0 (0)
**History of Physical Illness**					
None	16 (42.1)	12 (54.5)	11 (61.1)	14 (38.9)	3 (50.0)
Yes	22 (57.9)	10 (45.5)	7 (38.9)	22 (61.1)	3 (50.0)
**Estimated Life Expectancy, median (IQR), m**	5.00 (4.00, 6.00)	5.00 (4.00, 6.00)	5.00 (4.00, 6.00)	5.00 (4.00, 6.00)	4.50 (4.00, 5.25)
**Number of Past Physical Illnesses, median (IQR)**	1.00 (0.00, 1.00)	1.00 (0.00, 1.00)	0.00 (0.00, 1.25)	1.00 (0.00, 1.00)	0.50 (0.00, 1.00)
**VAS-Closeness Score, mean (SD)[a]**	6.89±1.72	6.68±1.64	6.83±1.43	6.92±1.89	6.17±0.98

Tab. S2: Demographic and Clinical Characteristics Across Specific Anxiety Trajectories (N = 60).

**Characteristics**	**Participants, No. (%)**					
	**Low-level, continuously increasing pre-death anxiety (n = 23)**	**Moderate-level, continuously increasing pre-death anxiety (n = 30)**	**High-level, rapidly increasing pre-death anxiety (n = 7)**	**High-level, rapidly decreasing post-death anxiety (n = 11)**	**Low-level, continuously decreasing post-death anxiety (n = 30)**	**High-level, continuously decreasing post-death anxiety (n = 19)**
**Sex**						
Female	9 (39.1)	19 (63.3)	4 (57.1)	5 (45.5)	14 (46.7)	13 (68.4)
Male	14 (60.9)	11 (36.7)	3 (42.9)	6 (54.5)	16 (53.3)	6 (31.6)
**Age, y**	52.91±10.99	51.37±9.75	58.71±8.08	53.36±4.72	50.53±10.99	56.11±10.60
20–40	3 (13.0)	2 (6.7)	0 (0)	0 (0)	4 (13.3)	1 (5.3)
41–60	13 (56.5)	25 (83.3)	4 (57.1)	10 (90.9)	20 (66.7)	12 (63.2)
61–80	7 (30.4)	3 (10.0)	3 (42.9)	1 (9.1)	6 (20.0)	6 (31.6)
**Ethnicity (Han)**	23 (100)	30 (100)	7 (100)	11 (100)	30 (100)	19 (100)
**Education Level**						
Illiterate	0 (0)	0 (0)	0 (0)	0 (0)	0 (0)	0 (0)
Primary School	2 (8.7)	4 (13.3)	1 (14.3)	1 (9.1)	4 (13.3)	2 (10.5)
Middle School	6 (26.1)	5 (16.7)	1 (14.3)	1 (9.1)	6 (20.0)	5 (26.3)
High School/Vocational School	9 (39.1)	5 (16.7)	1 (14.3)	2 (18.2)	9 (30.0)	4 (21.1)
Associate Degree	3 (13.0)	9 (30.0)	3 (42.9)	6 (54.5)	5 (16.7)	4 (21.1)
University and Above	3 (13.0)	7 (23.3)	1 (14.3)	1 (9.1)	6 (20.0)	4 (21.1)
**Residence**						
Rural	0 (0)	0 (0)	0 (0)	0 (0)	0 (0)	0 (0)
Urban	23 (100)	30 (100)	7 (100)	11 (100)	30 (100)	19 (100)
**Monthly Household Income (CNY)**						
1,000–2,000	23 (100)	30 (100)	7 (100)	4 (36.4)	5 (16.7)	4 (21.1)
2,000–5,000	23 (100)	30 (100)	7 (100)	0 (0)	14 (46.7)	8 (42.1)
5,000–10,000	23 (100)	30 (100)	7 (100)	7 (63.6)	11 (36.7)	7 (36.8)
**Dying being the Primary Financial Provider**						
No	16 (69.6)	15 (50.0)	2 (28.6)	5 (45.5)	18 (60.0)	10 (52.6)
Yes	7 (30.4)	15 (50.0)	5 (71.4)	6 (54.5)	12 (40.0)	9 (47.4)
**Marital Status**						
Unmarried	1 (4.3)	2 (6.7)	0 (0)	0 (0)	2 (6.7)	1 (5.3)
Married	21 (91.3)	28 (93.3)	5 (71.4)	11 (100.0)	27 (90.0)	16 (84.2)
Divorced	1 (4.3)	0 (0)	1 (14.3)	0 (0)	1 (3.3)	1 (5.3)
Widowed	0 (0)	0 (0)	1 (14.3)	0 (0)	0 (0)	1 (5.3)
**Drinking History**						
No	16 (69.6)	22 (73.3)	5 (71.4)	7 (63.6)	22 (73.3)	14 (73.7)
Yes	7 (30.4)	8 (26.7)	2 (28.6)	4 (36.4)	8 (26.7)	5 (26.3)
**Smoking History**						
No	10 (43.5)	17 (56.7)	4 (57.1)	5 (45.5)	15 (50.0)	11 (57.9)
Yes	13 (56.5)	13 (43.3)	3 (42.9)	6 (54.5)	15 (50.0)	8 (42.1)

**Substance Abuse History (No)**	23 (100.0)	30 (100.0)	7 (100.0)	11 (100.0)	30 (100.0)	19 (100.0)
**Family History of Psychiatric Illness (No)**	23 (100.0)	30 (100.0)	7 (100.0)	11 (100.0)	30 (100.0)	19 (100.0)
**Relationship with Terminally Ill Patient**						
Spouse	3 (13.0)	3 (10.0)	0 (0)	1 (9.1)	3 (10.0)	2 (10.5)
Offspring	18 (78.2)	21 (70.0)	5 (71.4)	8 (72.7)	22 (73.3)	14 (73.7)
Other (foster parent/child)	2 (8.7)	6 (20.0)	2 (28.6)	2 (18.2)	5 (16.7)	3 (15.8)
**Primary Diagnosis of Terminally Ill Patient**						
Respiratory System	5 (21.7)	4 (13.3)	0 (0)	1 (9.1)	6 (20.0)	2 (10.5)
Digestive System	0 (0)	2 (6.7)	0 (0)	0 (0)	2 (6.7)	0 (0)
Cardiovascular System	2 (8.7)	7 (23.3)	2 (28.6)	4 (36.4)	3 (10.0)	4 (21.1)
Cerebrovascular System	9 (39.1)	8 (26.7)	3 (42.9)	3 (27.3)	9 (30.0)	8 (42.2)
Nervous System	5 (21.7)	9 (30.0)	0 (0)	2 (18.2)	8 (26.7)	4 (21.1)
Urogenital System	0 (0)	0 (0)	1 (14.3)	0 (0)	0 (0)	1 (5.3)
Other	2 (8.7)	0 (0)	1 (14.3)	1 (9.1)	2 (6.7)	0 (0)
**History of Physical Illness**						
None	9 (39.1)	14 (46.7)	5 (71.4)	7 (63.6)	10 (33.3)	11 (57.9)
Yes	14 (60.9)	16 (53.3)	2 (28.6)	4 (36.4)	20 (66.7)	8 (42.1)
**Estimated Life Expectancy, median (IQR), m**	5.00 (4.00, 6.00)	5.00 (4.00, 6.00)	5.00 (4.00, 5.00)	6.00 (5.00, 6.00)	5.00 (3.75, 6.00)	5.00 (4.00, 6.00)
**Number of Past Physical Illnesses, median (IQR)**	1.00 (0.00, 1.00)	1.00 (0.00, 1.00)	0.00 (0.00, 1.00)	0.00 (0.00, 2.00)	1.00 (0.00, 1.00)	0.00 (0.00, 1.00)
**VAS-Closeness Score, mean (SD)[a]**	7.26±1.29	6.63±1.96	6.14±1.35	6.55±1.75	6.90±1.56	6.84±1.89

Tab. S3: Demographic and Clinical Characteristics Across Specific Stress Trajectories (N = 60).

**Characteristics**	**Participants, No. (%)**					
	**Persistently low-level pre-death stress (n = 35)**	**High-level continuously increasing pre-death stress (n = 25)**	**Low-level, continuously decreasing post-death stress (n = 10)**	**Moderate-level, rapidly decreasing post-death stress (n = 35)**	**High-level, rapidly decreasing post-death stress (n = 4)**	**Persistently high-level post-death stress (n = 11)**
**Sex**						
Female	16 (45.7)	16 (64.0)	2 (20.0)	21 (60.0)	1 (25.0)	8 (72.7)
Male	19 (54.3)	9 (36.0)	8 (80.0)	14 (40.0)	3 (75.0)	3 (27.3)
**Age, y**	52.20±10.76	53.68±9.45	52.80±10.32	52.74±9.90	57.25±3.95	51.45±12.94
20–40	4 (11.4)	1 (4.0)	1 (10.0)	2 (5.7)	0 (0)	2 (18.2)
41–60	23 (65.7)	19 (76.0)	6 (60.0)	27 (77.1)	3 (75.0)	6 (54.5)
61–80	8 (22.9)	5 (20.0)	3 (30.0)	6 (17.1)	1 (25.0)	3 (27.3)
**Ethnicity (Han)**	35 (100)	25 (100)	10 (100)	35 (100)	4 (100)	11 (100)
**Education Level**						
Illiterate	0 (0)	0 (0)	0 (0)	0 (0)	0 (0)	0 (0)
Primary School	3 (8.6)	4 (16.0)	1 (10.0)	6 (17.1)	0 (0)	0 (0)
Middle School	8 (22.9)	4 (16.0)	0 (0)	8 (22.9)	1 (25.0)	3 (27.3)
High School/Vocational School	9 (25.7)	6 (24.0)	4 (40.0)	8 (22.9)	2 (50.0)	1 (9.1)
Associate Degree	9 (25.7)	6 (24.0)	4 (40.0)	7 (20.0)	1 (25.0)	3 (27.3)
University and Above	6 (17.1)	5 (20.0)	1 (10.0)	6 (17.1)	0 (0)	4 (36.4)
**Residence**						
Rural	0 (0)	0 (0)	0 (0)	0 (0)	0 (0)	0 (0)
Urban	35 (100)	25 (100)	10 (100)	35 (100)	4 (100)	11 (100)
**Monthly Household Income (CNY)**						
1,000–2,000	8 (22.9)	5 (20.0)	2 (20.0)	8 (22.9)	1 (25.0)	2 (18.2)
2,000–5,000	13 (37.1)	9 (36.0)	3 (30.0)	14 (40.0)	2 (50.0)	3 (27.3)
5,000–10,000	14 (40.0)	11 (44.0)	5 (50.0)	13 (37.1)	1 (25.0)	6 (54.5)
**Dying being the Primary Financial Provider**						
No	20 (57.1)	13 (52.0)	5 (50.0)	20 (57.1)	2 (50.0)	6 (54.5)
Yes	15 (42.9)	12 (48.0)	5 (50.0)	15 (42.9)	2 (50.0)	5 (45.5)
**Marital Status**						
Unmarried	2 (5.7)	1 (4.0)	0 (0)	1 (2.9)	0 (0)	2 (18.2)
Married	31 (88.6)	23 (92.0)	9 (90.0)	33 (94.3)	4 (100.0)	8 (72.7)
Divorced	1 (2.9)	1 (4.0)	1 (10.0)	0 (0)	0 (0)	1 (9.1)
Widowed	1 (2.9)	0 (0)	0 (0)	1 (2.9)	0 (0)	0 (0)
**Drinking History**						
No	26 (74.3)	17 (68.0)	6 (60.0)	26 (74.3)	2 (50.0)	9 (81.8)
Yes	9 (25.7)	8 (32.0)	4 (40.0)	9 (25.7)	2 (50.0)	2 (18.2)
**Smoking History**						
No	17 (48.6)	14 (56.0)	3 (30.0)	20 (57.1)	2 (50.0)	6 (54.5)
Yes	18 (51.4)	11 (44.0)	7 (70.0)	15 (42.9)	2 (50.0)	5 (45.5)
**Substance Abuse History (No)**	35 (100.0)	25 (100.0)	10 (100.0)	35 (100.0)	4 (100.0)	11 (100.0)

**Family History of Psychiatric Illness (No)**	35 (100.0)	25 (100.0)	10 (100.0)	35 (100.0)	4 (100.0)	11 (100.0)
**Relationship with Terminally Ill Patient**						
Spouse	4 (11.4)	2 (8.0)	1 (10.0)	3 (8.6)	0 (0)	2 (18.2)
Offspring	25 (71.4)	19 (76.0)	9 (90.0)	24 (68.6)	4 (100.0)	7 (63.6)
Other (foster parent/child)	6 (17.1)	4 (16.0)	0 (0)	8 (22.9)	0 (0)	2 (18.2)
**Primary Diagnosis of Terminally Ill Patient**						
Respiratory System	8 (22.9)	1 (4.0)	3 (30.0)	4 (11.4)	0 (0)	2 (18.2)
Digestive System	1 (2.9)	1 (4.0)	0 (0)	1 (2.9)	0 (0)	1 (9.1)
Cardiovascular System	6 (17.1)	5 (20.0)	1 (10.0)	6 (17.1)	1 (25.0)	3 (27.3)
Cerebrovascular System	11 (31.4)	9 (36.0)	3 (30.0)	11 (31.4)	3 (75.0)	3 (27.3)
Nervous System	6 (17.1)	8 (32.0)	2 (20.0)	10 (28.6)	0 (0)	2 (18.2)
Urogenital System	1 (2.9)	0 (0)	0 (0)	1 (2.9)	0 (0)	0 (0)
Other	2 (5.7)	1 (4.0)	1 (10.0)	2 (5.7)	0 (0)	0 (0)
**History of Physical Illness**						
None	16 (45.7)	12 (48.0)	5 (50.0)	14 (40.0)	3 (75.0)	6 (54.5)
Yes	19 (54.3)	13 (52.0)	5 (50.0)	21 (60.0)	1 (25.0)	5 (45.5)
**Estimated Life Expectancy, median (IQR), m**	5.00 (4.00, 6.00)	5.00 (4.00, 6.00)	6.00 (5.00, 6.00)	5.00 (4.00, 6.00)	5.00 (4.00, 6.00)	5.00 (4.00, 6.00)
**Number of Past Physical Illnesses, median (IQR)**	1.00 (0.00, 1.00)	1.00 (0.00, 1.00)	0.50 (0.00, 1.00)	1.00 (0.00, 1.00)	0.00 (0.00, 2.25)	0.00 (0.00, 1.00)
**VAS-Closeness Score, mean (SD)[a]**	7.09±1.65	6.44±1.69	7.20±1.40	6.69±1.80	7.50±0.58	6.64±1.86
